# Supplementary material for: The Choice of Anti-Inflammatory Influences the Elimination of Protein-Bound Uremic Toxins
Source: Toxins (Basel). 2024 Dec 16;16(12):545. doi: 10.3390/toxins16120545 (PMC11679929; doi:10.3390/toxins16120545)
Supplement: Supplementary file 1 [file toxins-16-00545-s001.zip › toxins-3337680-supplementary.pdf]

Supplementary Materials: The Choice of Anti-Inflammatory Influences the Elimination of Protein-Bound Uremic Toxins

Víctor Joaquín Escudero-Saiz, Elena Cuadrado-Payán, María Rodriguez-Garcia, Gregori Casals, Lida María Rodas, Néstor Fontseré, María del Carmen Salgado, Carla Bastida, Nayra Rico, José Jesús Broseta and Francisco Maduell

Table S1. Individual values of indoxyl sulfate and p-cresyl sulfate concentrations in serum pre and post-dialysis.

	Lysine acetylsalicylic						Acetaminophen						Dexketoprofen						Ibuprofen					
	Indoxyl sulfate			p-cresyl sulfate			Indoxyl sulfate			p-cresyl sulfate			Indoxyl sulfate			p-cresyl sulfate			Indoxyl sulfate			p-cresyl sulfate		
Patient	Pre-dialysis	Post-dialysis	RR (%)	Pre-dialysis	Post-dialysis	RR (%)	Pre-dialysis	Post-dialysis	RR (%)	Pre-dialysis	Post-dialysis	RR (%)	Pre-dialysis	Post-dialysis	RR (%)	Pre-dialysis	Post-dialysis	RR (%)	Pre-dialysis	Post-dialysis	RR (%)	Pre-dialysis	Post-dialysis	RR (%)
1	41331	21406	48,2	63122	30651	51,4	42733	22505	47,3	59594	31758	46,7	47762	23835	50,1	73720	38876	47,3	47102	22985	51,2	63146	32737	48,2
2	21399	9473	55,7	54438	27967	48,6	17987	8314	53,8	48634	25792	47,0	14817	6659	55,1	36243	19149	47,2	15255	6739	55,8	25975	13053	49,7
3	24107	11146	53,8	74990	35969	52,0	35407	17387	50,9	71111	37030	47,9	26533	11662	56,0	82995	37694	54,6	28538	10483	63,3	49161	19782	59,8
4	32105	18070	43,7	24641	16752	32,0	34630	15360	55,6	23511	11880	49,5	28487	13336	53,2	26676	13649	48,8	31756	12887	59,4	21856	9852	54,9
5	19906	7432	62,7	39918	15652	60,8	23146	9294	59,8	43061	18105	58,0	32205	12242	62,0	56819	23163	59,2	36912	11370	69,2	54471	17364	68,1
6	35124	20003	43,1	28104	16197	42,4	24177	13269	45,1	26575	16595	37,6	21332	11256	47,2	17906	10235	42,8	27438	12654	53,9	27440	13586	50,5
7	41544	25904	37,6	18355	12933	29,5	47486	31606	33,4	21698	15456	28,8	47106	30827	34,6	23338	16353	29,9	36940	19847	46,3	14346	8797	38,7
8	39643	22608	43,0	24651	15797	35,9	38996	21361	45,2	23822	14917	37,4	36823	18872	48,7	24941	14772	40,8	41963	19512	53,5	24049	12171	49,4
9	61916	28965	53,2	52271	24405	53,3	50218	24116	52,0	32557	17648	45,8	46384	22909	50,6	40206	21755	45,9	51204	22926	55,2	43705	24151	44,7
10	7862	4304	45,2	50113	26906	46,3	7728	4066	47,4	47642	24066	49,5	6177	3026	51,0	51371	23967	53,3	11584	5037	56,5	48688	23548	51,6
11	31680	14660	53,7	22327	11407	48,9	32904	13972	57,5	15169	6558	56,8	34231	16284	52,4	37297	20052	46,2	39007	11910	69,5	22030	8048	63,5
12	20097	10710	46,7	31066	17050	45,1	28305	13882	51,0	34902	18483	47,0	28924	14348	50,4	26621	14385	46,0	28333	11846	58,2	30677	14777	51,8
13	41560	16441	60,4	29624	13075	55,9	37318	21059	43,6	27957	16865	39,7	44651	22083	50,5	42225	22396	47,0	36349	14969	58,8	41738	18960	54,6
14	16070	5820	63,8	67993	26280	61,3	16618	7095	57,3	74670	32088	57,0	14650	6254	57,3	67555	29437	56,4	14701	4966	66,2	77048	25867	66,4
15	12333	6324	48,7	44986	25097	44,2	9603	4468	53,5	33815	17890	47,1	13598	6354	53,3	40953	21340	47,9	10686	4262	60,1	36650	16053	56,2
16	43435	21813	49,8	29619	16683	43,7	45768	25680	43,9	25549	16100	37,0	48454	25034	48,3	30145	17651	41,4	44629	21974	50,8	28833	15507	46,2
17	6013	1936	67,8	4414	1553	64,8	4065	1380	66,1	2689	914	66,0	4001	1244	68,9	2722	677	75,1	3966	1156	70,8	2240	446	80,1
18	35763	12565	64,9	26156	10750	58,9	40385	13878	65,6	37438	13887	62,9	34212	12280	64,1	28235	11775	58,3	33927	9490	72,0	21188	6278	70,4
19	41960	18452	56,0	33140	14993	54,8	35076	16989	51,6	29178	15405	47,2	43713	25001	42,8	44071	26626	39,6	38953	17489	55,1	43556	21124	51,5
20	22563	13015	42,3	22254	13163	40,8	21674	11987	44,7	21454	12057	43,8	25371	14316	43,6	12157	7359	39,5	24445	12043	50,7	12328	7075	42,6
21	42287	19792	53,2	47315	24857	47,5	39577	17777	55,1	55190	26230	52,5	42272	21335	49,5	50692	23676	53,3	49320	19711	60,0	56241	23882	57,5
22	12928	6379	50,7	8174	3939	51,8	14932	5041	66,2	21202	8499	59,9	15023	6927	53,9	19800	10109	48,9	16053	5334	66,8	22362	8716	61,0
23	13674	4548	66,7	6250	2257	63,9	14746	4410	70,1	8279	2767	66,6	11171	3514	68,5	4618	1512	67,3	11766	3426	70,9	4601	1469	68,1

RR: reduction ratio. Note all post-dialysis values are corrected for the degree of hemoconcentration and the volume of distribution (approximate extracellular volume) according to Bergström and Wehle [37]. IS and pCS values are measured in ng/mL.
